# Gankyrin promotes epithelial-mesenchymal transition and metastasis in NSCLC through forming a closed circle with IL-6/ STAT3 and TGF-β/SMAD3 signaling pathway

**DOI:** 10.18632/oncotarget.13947

**Published:** 2016-12-15

**Authors:** Wu-ping Wang, Ying Sun, Qiang Lu, Jin-bo Zhao, Xue-jiao Wang, Zhao Chen, Yun-feng Ni, Ju-zheng Wang, Yong Han, Zhi-pei Zhang, Xiao-long Yan, Xiao-fei Li

**Affiliations:** ^1^ Department of Thoracic Surgery, Tangdu Hospital, The Fourth Military Medical University, Xi’an, 710038, China

**Keywords:** NSCLC, gankyrin, EMT, metastasis, closed circle

## Abstract

Our previous research showed that Gankyrin was overexpressed in NSCLC and significantly associated with clinicopathologic features and poor prognosis. In this study, we will explore potential effect of Gankyrin on EMT and metastasis in NSCLC. The ectopic higher expression of Gankyrin markedly increased the migration and invasion in NSCLC cells. In contrast, silencing Gankyrin inhibit this aggressive behavior in NSCLC cells. Further study demonstrated that overexpression of Gankyrin could decrease E-cadherin expression and increase expression of Vimentin and Twist1 at mRNA and protein levels. These data indicated that Gankyrin could facilitate occurrence and development of EMT. Also IHC analysis showed that Gankyrin expression was negatively correlated with E-cadherin expression, while positively correlated with Vimentin and Twist1 expression in NSCLC tissues. The mechanism study finally suggested that the Gankyrin-driven EMT was partially due to IL-6/p-STAT3 and TGF-β/p-SMAD3 pathways activation. Taken together, our data provided a novel mechanism of Gankyrin promoting EMT and metastasis in NSCLC through forming a closed circle with IL-6/p-STAT3 and TGF-β/p-SMAD3 signaling pathway.

## INTRODUCTION

Lung cancer is responsible for approximately 1.4 million deaths annually [1, 2]. According to the World Health Organization (WHO)'s estimates, more than one million Chinese will be diagnosed with lung cancer every year by 2025, thus the death toll will increase accordingly [3]. In lung cancer, approximately 85% are non-small cell lung cancer (NSCLC) [4], and a majority of NSCLC patients die from local recurrence or distant metastasis even after undergoing curative surgical resection, thus the 5-year survival rate is still unsatisfactory [5]. Therefore, there is an urgent requirement to understand the metastatic mechanism in NSCLC for targeted therapy and prognosis.

Since the report of the pivotal role of the epithelial-mesenchymal transition (EMT) during embryonic development in the 1980s, many studies have demonstrated that EMT plays essential roles in tumor progression, especially in tumor metastasis [6, 7]. In lung cancer, the EMT has been recognized as an initial step and critical procedure of tumor cell metastasis [8–10]. Signaling pathways, such as IL-6/STAT3, TGF-β/SMAD3, PI3K/AKT and et al, can induce EMT during development and differentiation [11, 12]. However, the mechanisms underlying EMT induction in cancer cells remain unclear.

Gankyrin (also known as PSMD 10 or p28GANK) was initially purified and characterized by Tanaka and coworkers in 1998 [13]. Recently, it was identified as an oncoprotein frequently overexpressed in several cancers [14–19]. We also tested Gankyrin expression in NSCLC in preliminary study [20]. The results showed that the gene and protein of Gankyrin expressed higher in human NSCLC tissues than in the corresponding adjacent non-cancerous tissues, and Gankyrin overexpression was significantly associated with lymph node metastasis and poor prognosis. These data indicated that Gankyrin might be a conservative molecule playing essential roles in tumor progression. However, until now, the exact mechanisms of Gankyrin in NSCLC progression still remain unknown. Our previous study also showed an apparent prognostic significance of Gankyrin overexpression in early stage patients, but no prognostic significance in late stage patients [20], which suggested that Gankyrin might participate in the initial step of NSCLC metastasis, such as the EMT process.

Both the IL-6 and TGF-β are important cytokines initiating EMT in tumor microenvironment. However, once the tumor cells acquire the EMT phenotype induced by IL-6 and TGF-β, they leave away from the primary sites of the microenvironment. The concentrations of those cytokines surrounding the cells reduce rapidly, so what are the mechanisms for the cells to sustain the EMT phenotypes till they migrate and locate in a new metastatic site. We guess that there are some key proteins to connect the downstream signal molecules of IL-6 and TGF-β, such as phosphorylated STAT3 (p-STAT3) and phosphorylated SMAD3 (p-SAMD3), forming a closed circle to sustain the EMT phenotype [21, 22]. Our preliminary experimental results show that Gankyrin expression was correlated with IL-6 and TGF-β, so we speculate that Gankyrin may be one of them.

In the present study, we demonstrated that Gankyrin was upregulated in NSCLC tissues and cell lines. Over-expressed Gankyrin increases NSCLC migration and invasion, and silencing Gankyrin inhibited NSCLC migration and invasion. Further study demonstrated that over-expression of Gankyrin could decrease expression of E-cadherin and increase expression of Vimentin and Twist1, and silencing Gankyrin expression could increase expression of E-cadherin and decrease expression of Vimentin and Twist1. Furthermore, our study provided a new mechanism that Gankyrin-promoted EMT and metastasis in NSCLC was partially due to forming a closed circle with IL-6/p-STAT3 and TGF-β/p-SMAD3 signaling pathways.

## RESULTS

### Gankyrin was over-expressed in human NSCLC cell lines

**Figure 1:**
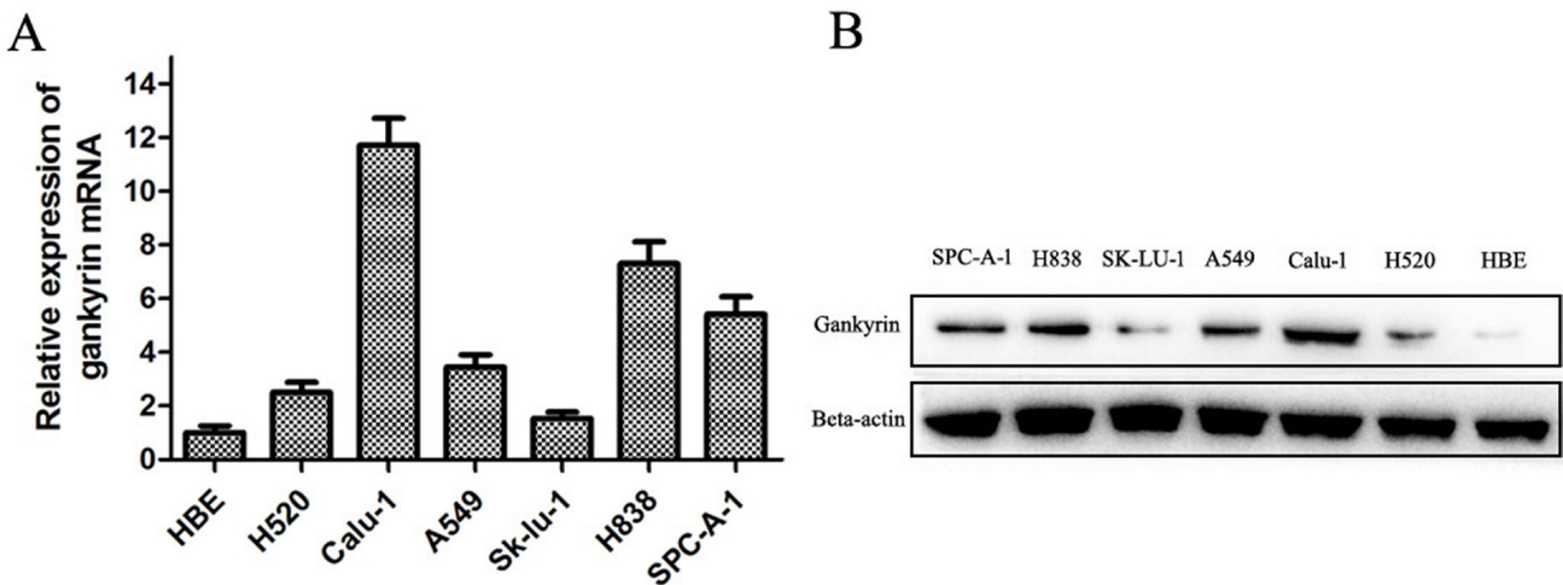
Gankyrin mRNA and protein expressions in six human NSCLC and normal bronchial epithelium cell lines (**A**) Gankyrin mRNA expression was higher in human NSCLC cell lines than HBE cell line. (**B**) Gankyrin protein expression was higher in human NSCLC cell lines than HBE cell line.

We assessed Gankyrin expression in six human NSCLC cell lines (A549, Sk-lu-1, SPC-A-1, H838, H520, Calu-1) and one normal human bronchial epithelium cell line (HBE) using Semi-quantitative RT-PCR and Western blot assay. As shown in Figure 1A and 1B, Gankyrin expression was higher in the NSCLC cell lines (H520, Calu-1, A549, Sk-lu-1, H838, SPC-A-1) when compared with its expression in the HBE cell line both at the mRNA and protein levels.

### Silencing Gankyrin inhibited NSCLC cell migration and invasion

**Figure 2:**
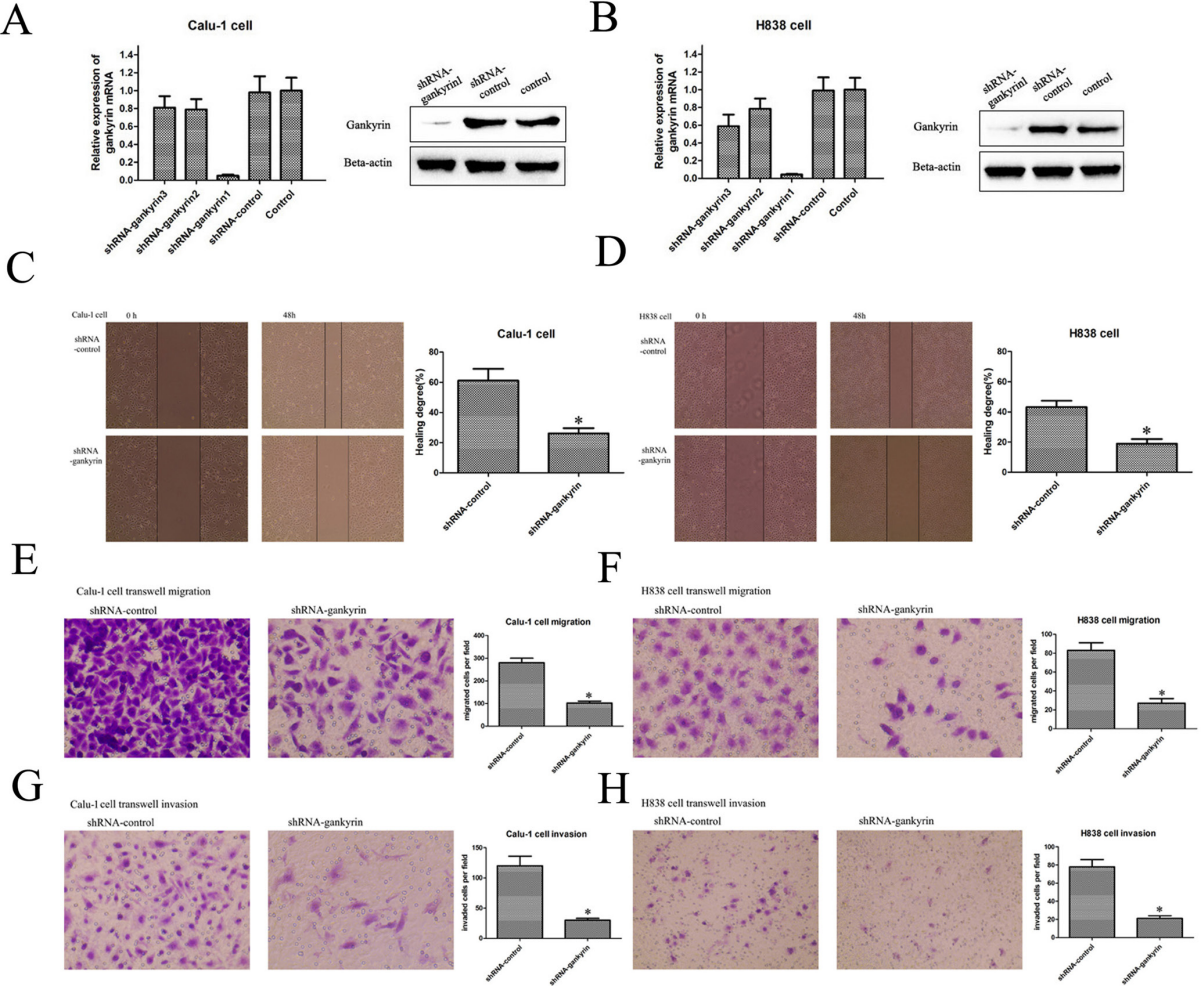
Silencing Gankyrin inhibited NSCLC cell migration and invasion (**A**) and (**B**) Lenti-virus transduction decreased the Gankyrin expression in Calu-1 and H838 cell lines. (**C**) and (**D**) Scratch wound healing assay showed that knockdown of Gankyrin inhibited the migration of Calu-1 and H838 cell lines. (**E**) and (**F**) Transwell migration assay showed that knockdown of Gankyrin inhibited the migration of Calu-1 and H838 cell lines. (**G**) and (**H**) Transwell assay showed that knockdown of Gankyrin inhibited the invasion of Calu-1 and H838 cell lines. *P < 0.05.

Our previous results revealed that high level of Gankyrin was associated with poor prognosis in NSCLC patients, but the underlying mechanisms remain unclear. Thus, to find out whether Gankyrin was associated with poor prognosis of NSCLC patients through promoting tumor metastasis, we applied lenti-virus transduction to knockdown the Gankyrin expression in Calu-1 and H838 cell lines (Figure 2A and 2B) and then detected the migratory and invasive ability of these cell lines, in which the Gankyrin expression was relatively higher than that in other NSCLC cell lines. The results suggested that the disruption of Gankyrin expression could significantly inhibit the migratory and invasive ability of Calu-1 and H838 cells by wounding healing (Figure 2C and 2D) and transwell migration (Figure 2E and 2F) and invasion assay (Figure 2G and 2H).

### Gankyrin overexpression could promote the NSCLC cell migration and invasion

**Figure 3:**
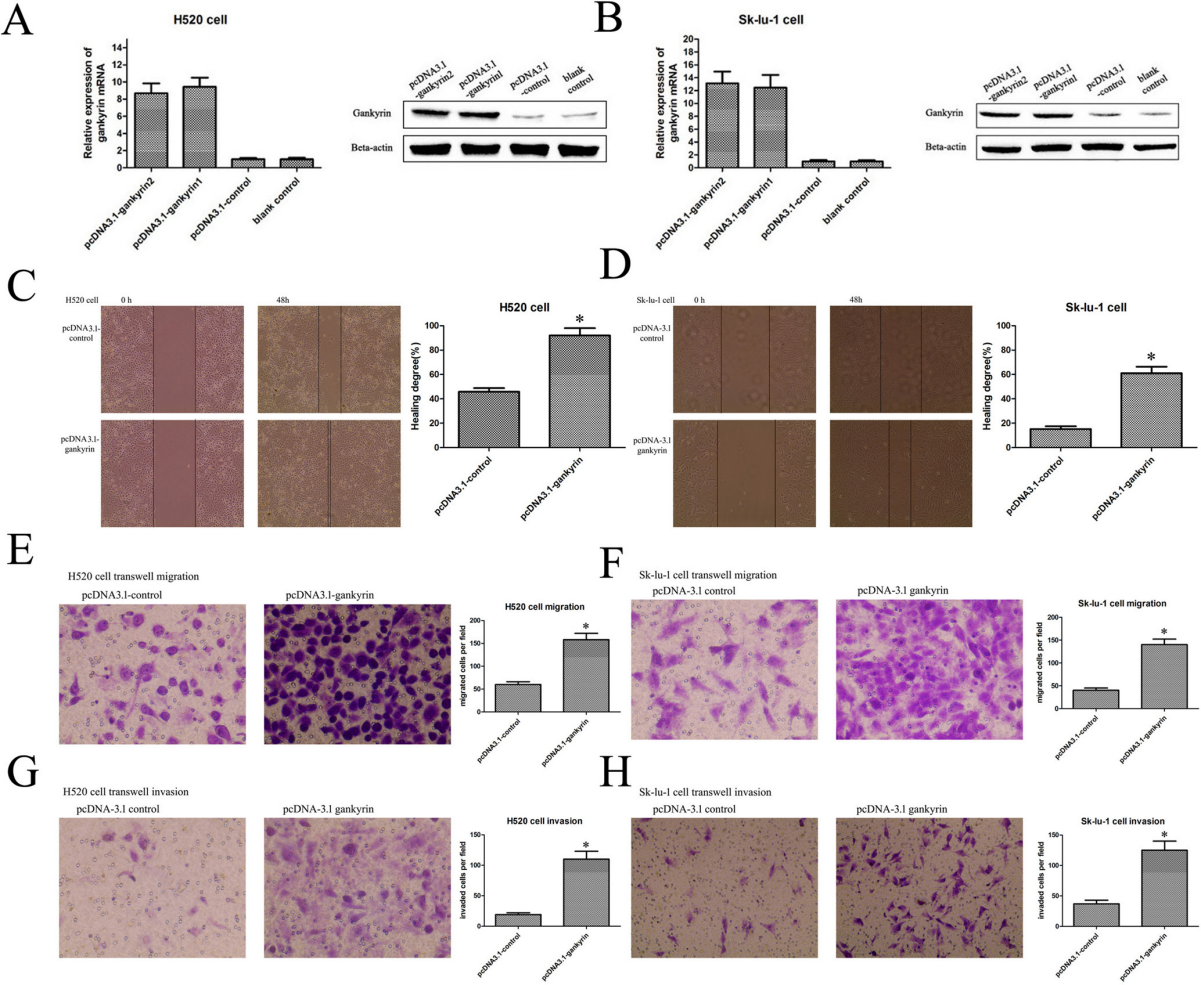
Gankyrin over-expression could promote the NSCLC cell migration and invasion (**A**) and (**B**) Plasmid transduction promoted the Gankyrin expression in H520 and SK-LU-1 cell lines. (**C**) and (**D**) Scratch wound healing assay showed that overexpession of Gankyrin promoted the migration of H520 and SK-LU-1 cell lines. (**E**) and (**F**) Transwell migration assay showed that overexpession of Gankyrin promoted the migration of H520 and SK-LU-1 cell lines. (**G**) and (**H**) Transwell assay showed that overexpession of Gankyrin promoted the invasion of H520 and SK-LU-1 cell lines. *P < 0.05.

To further prove the role of Gankyrin in NSCLC metastasis, we stably overexpressed Gankyrin expression in H520 and Sk-lu-1 cell lines, in which the Gankyrin expression is relatively lower than that in the other NSCLC cell lines (Figure 3A and 3B). Wounding healing and transwell assay showed that Gankyrin overexpression could significantly increase the migratory and invasive ability of H520 and Sk-lu-1 cells (Figure 3C–3H). These findings support the hypothesis that Gankyrin plays an important role in promoting NSCLC metastasis.

### Gankyrin could induce the EMT phenotypes in NSCLC cell lines

**Figure 4:**
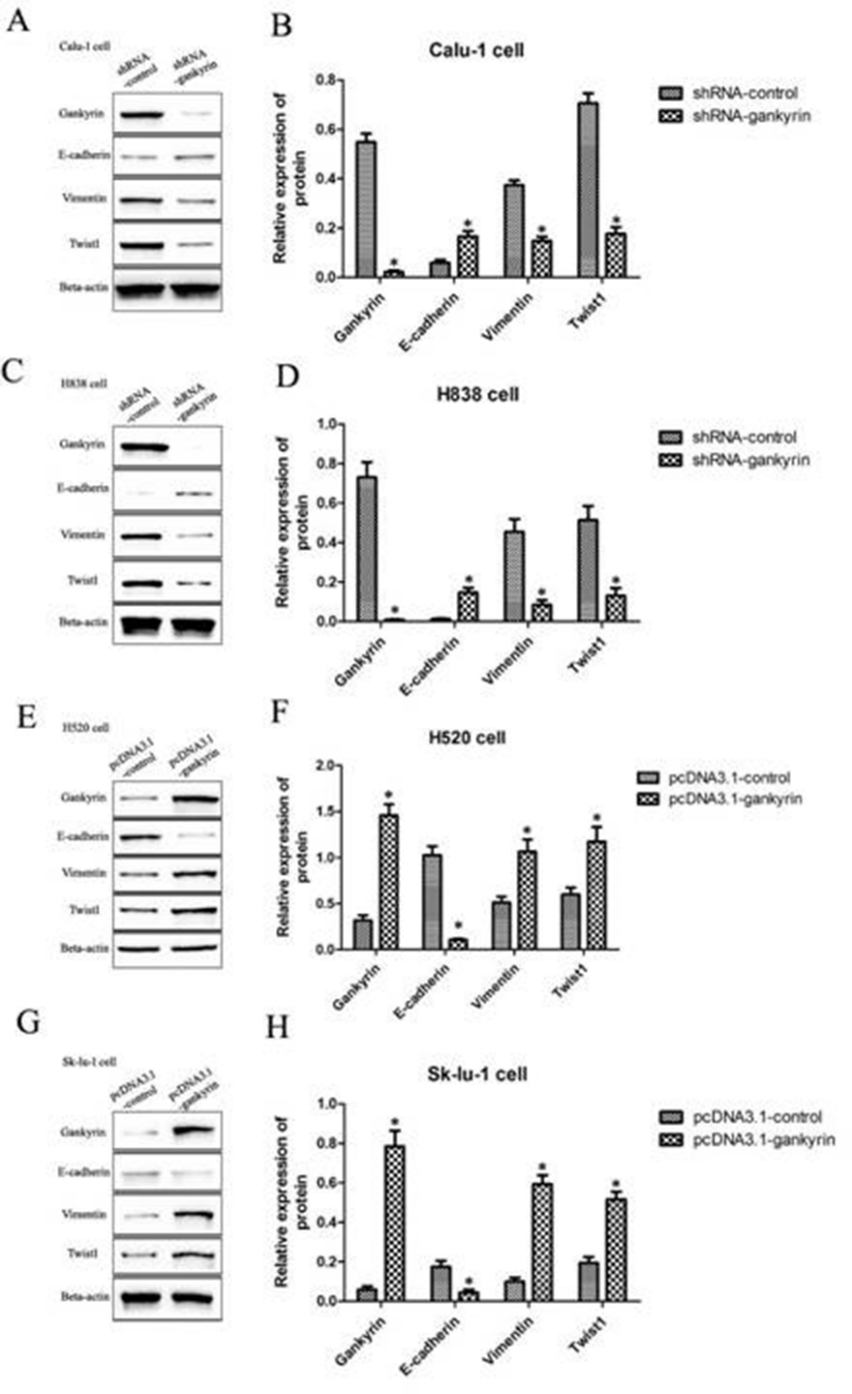
EMT-related biomarkers including E-cadherin, Vimentin and Twist1 were tested by qRT-PCR and western blot assays (**A**) and (**B**) E-cadherin, Vimentin and Twist1 mRNA and protein expressions in Gankyrin decreased Calu-1 cell line. (**C**) and (**D**) E-cadherin, Vimentin and Twist1 mRNA and protein expressions in Gankyrin decreased H838 cell line. (**E**) and (**F**) E-cadherin, Vimentin and Twist1 mRNA and protein expressions in Gankyrin increased H520 cell line. (**G**) and (**H**) E-cadherin, Vimentin and Twist1 mRNA and protein expressions in Gankyrin increased SK-LU-1 cell line. *P < 0.05.

**Figure 5:**
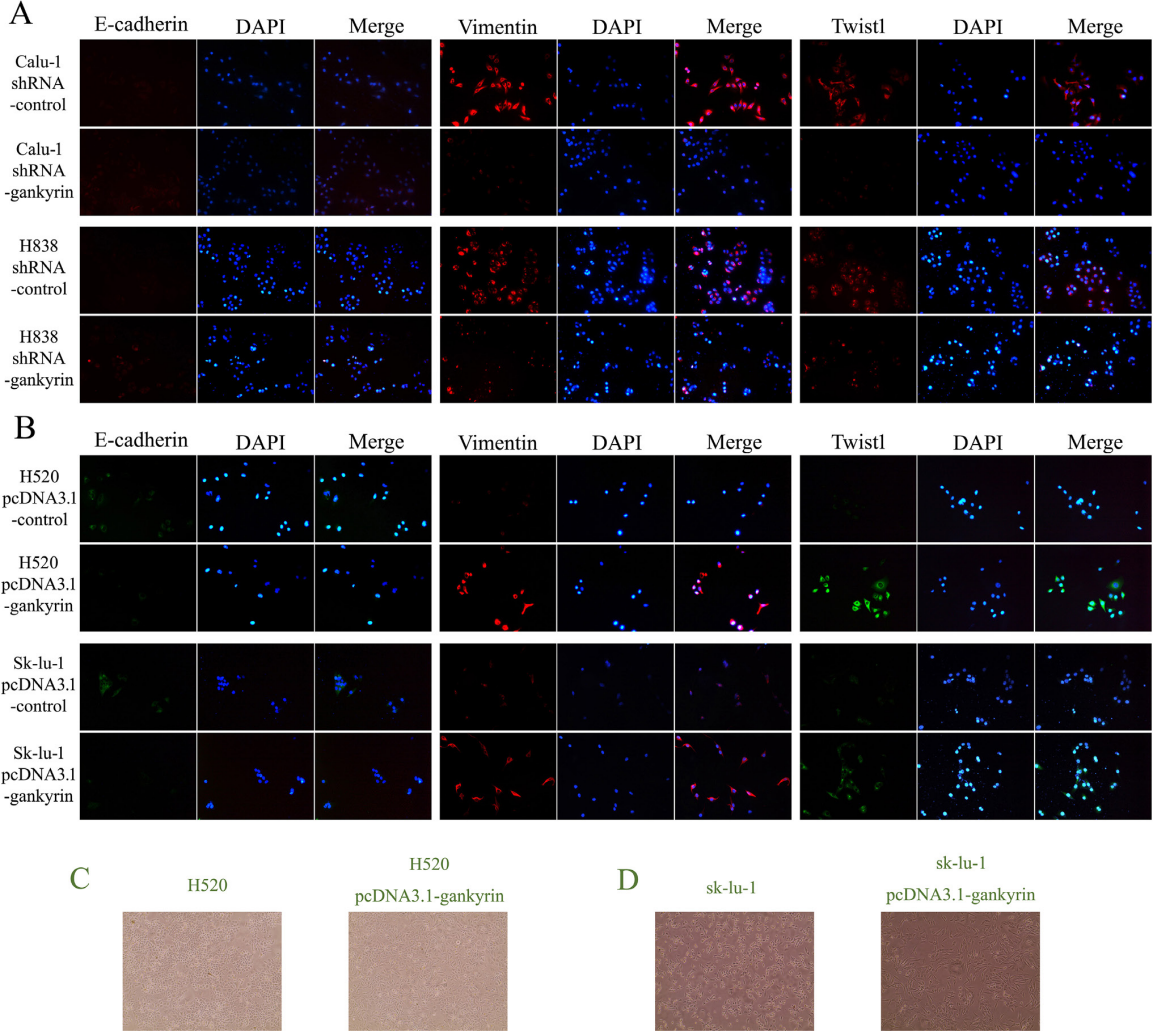
E-cadherin, Vimentin and Twist1 expression were detected both in Gankyrin decreased and increased NSCLC cell lines (**A**) The expression of E-cadherin, Vimentin and Twist1 in Gankyrin decreased NSCLC Calu-1 and H838 cell lines. (**B**) The expression of E-cadherin, Vimentin and Twist1 in Gankyrin increased NSCLC H520 and SK-LU-1 cell lines. (**C**) The changes of H520 cell shape when Gankyrin protein was stably overexpressed. (**D**) The changes of sk-lu-1 cell shape when Gankyrin protein was stably overexpressed.

To further explore potential mechanisms of Gankyrin associated with metastasis in NSCLC, essential EMT-related biomarkers including E-cadherin, Vimentin and Twist1 were tested by quantitative RT-PCR and Western blot assay. We found that lower mRNA and protein levels of mesenchymal markers were expressed in Gankyrin knockdown cells lines (Calu-1-shRNA-Gankyrin and H838-shRNA-Gankyrin), such as Vimentin and Twist1, whereas higher mRNA and protein levels of the epithelial marker of E-cadherin were observed (Figure 4A–4D). In addition, we observed increased expression of the epithelial marker E-cadherin and decreased expression of mesenchymal markers Vimentin, Twist in Gankyrin knockdown cells lines by immunoblotting assay (Figure 5A). In line with the above observations, when Gankyrin was stably overexpressed in H520 and Sk-lu-1 cells, we found that both H520 pCDNA3.1-Gankyrin cells and Sk-lu-1 pCDNA3.1-Gankyrin cells expressed higher mRNA and protein levels of mesenchymal markers, such as Vimentin and Twist1, whereas lower mRNA and protein levels of the epithelial marker of E-cadherin were observed (Figure 4E–4H). Immunoblotting assay in Gankyrin overexpressed cell lines also showed increased expression of the mesenchymal markers Vimentin, Twist1 and decreased expression of the epithelial marker E-cadherin (Figure 5B). Together, these observations showed that Gankyrin might induce EMT and metastasis in NSCLC cells.

### IHC results showed that Gankyrin expression correlated with EMT-related biomarkers in NSCLC tissues

**Figure 6:**
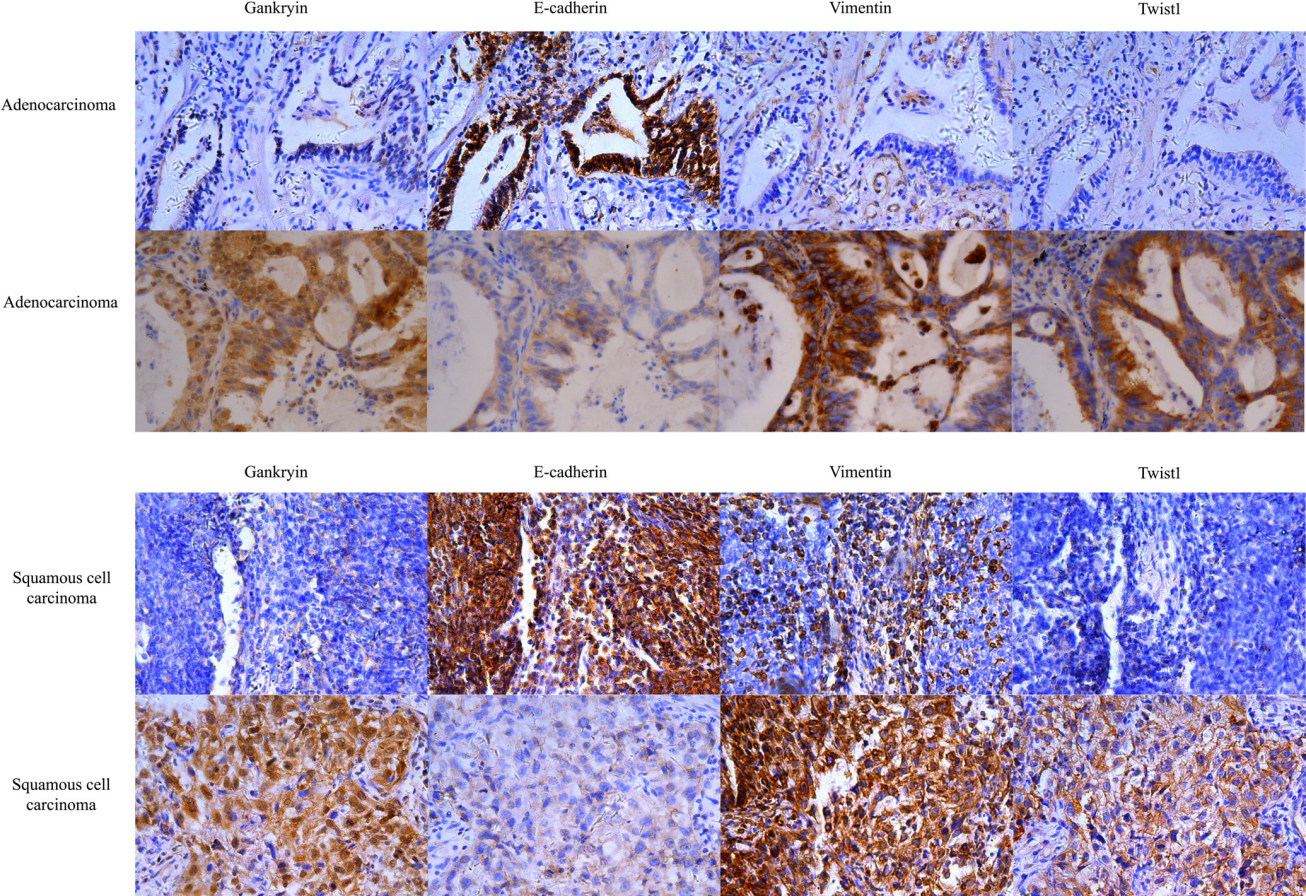
Gankyrin expression correlates with EMT-related biomarkers in NSCLC tissues (**A**) EMT-related biomarkers including E-cadherin, Vimentin and Twist1 were detected in ADC tissues using IHC. (**B**) EMT-related biomarkers including E-cadherin, Vimentin and Twist1 were detected in SCC tissues using IHC. ADC: Adenocarcinoma; SCC: Squamous cell carcinoma.

We used immunohistochemistry (IHC) to analyze the frequency of three EMT-related biomarkers (E-cadherin, Vimentin and Twist1) in the same investigation cohort as Gankyrin expression was studied. Representative microphotographs of the expression of these three biomarkers in ADC and SCC tissues are shown in Figure 6A and 6B. The percentage of positive expression of E-cadherin, Vimentin and Twist1 in Gankyrin-positive tumors and Gankyrin-negative tumors in ADC and SCC tissues were shown in Tables 1 and 2, respectively. Correlation analysis showed that Gankyrin expression in ADC or SCC tissues had positive correlations with the expression of Vimentin (*r* = 0.290, *P* = 0.027; *r* = 0.251 *P* = 0.017) and Twist1 (*r* = 0.347, *P* = 0.007; *r* = 0.247 *P* = 0.024), while negative correlations with the E-cadherin expression (*r* = –0.340, *P* = 0.009; *r* = –0.250, *P* = 0.018). These data strongly supported that Gankyrin was associated with EMT in NSCLC.

**Table 1:** Correlation of Gankyrin expression with E-cadherin, vimentin and twist1 in ADC tissues

ADC tissues	Gankyrin expression		Correlation coefficient	*P*-value
	Positive (%)	Negative (%)		
**E-cadherin expression**			*r* = –0.340	0.009
Positive (%)	23	8		
Negative (%)	36	1		
**Vimentin expression**			*r* = 0.290	0.027
Positive (%)	38	2		
Negative (%)	21	7		
**Twist1 expression**			*r* = 0.347	0.007
Positive (%)	42	3		
Negative (%)	17	6		

**Table 2:** Correlation of Gankyrin expression with E-cadherin, vimentin and twist1 in SCC tissues

SCC tissues	Gankyrin expression		Correlation coefficient	*P*-value
	Positive (%)	Negative (%)		
**E-cadherin expression**			*r* = –0.250	0.018
Positive (%)	34	18		
Negative (%)	40	6		
**Vimentin expression**			*r* = 0.251	0.017
Positive (%)	49	9		
Negative (%)	25	15		
**Twist1 expression**			*r* = 0.247	0.024
Positive (%)	54	11		
Negative (%)	20	13		

### Gankyrin-promoted EMT is partially due to IL-6/p-STAT3 and TGF-β/p-SMAD3 pathways activation

**Figure 7:**
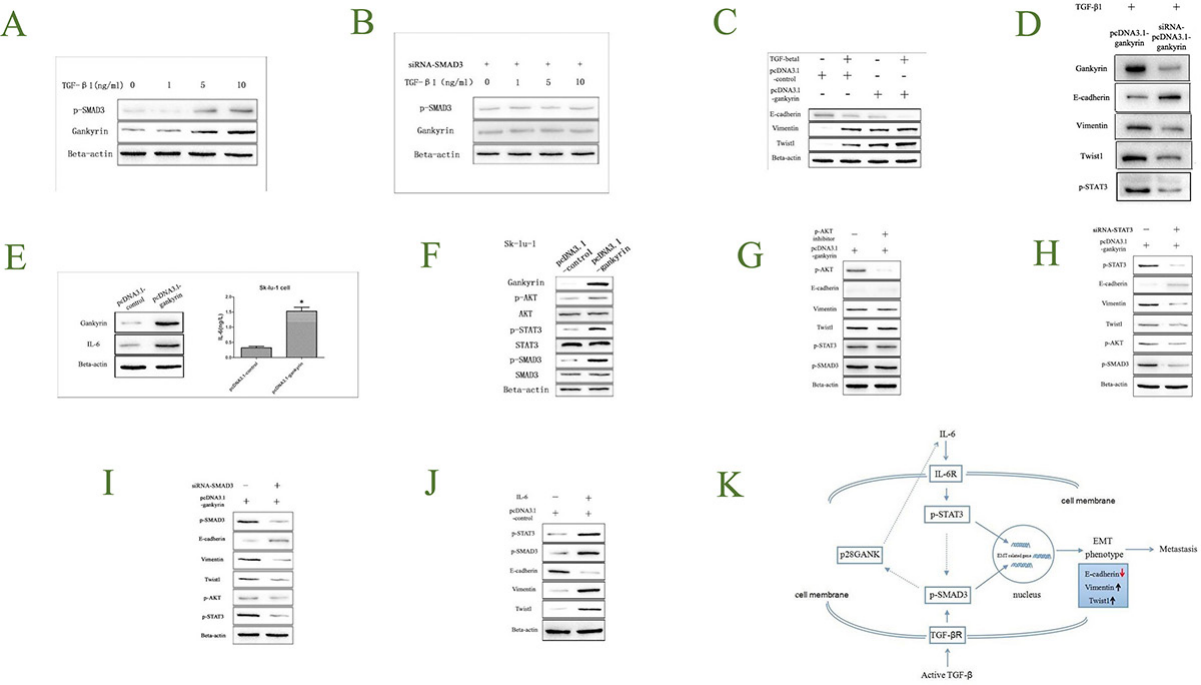
Gankyrin-promoted EMT is partially due to IL-6/p-STAT3 and TGF-β1/p-SMAD3 pathway activation (**A**) TGF-β1 could regulate p-SMAD3 and Gankyrin expressions. (**B**) TGF-β1 could regulate Gankyrin expression through p-SMAD3. (**C**) Gankyrin promotes the process of TGF-β1 induced EMT. (**D**) Silencing Gankyrin inhibits EMT and p-STAT3 induce by TGF-β1. (**E**) Gankyrin over-expression could up-regulate IL-6 level. (**F**) Gankyrin could increase p-AKT, p-STAT3, p-SMAD3 expressions. (**G**) The process of TGF-β1 induced EMT has nothing to do with p-AKT. (**H**–**J**) STAT3 and SMAD3 could promote the process of Gankyrin induced EMT. (**K**) IL-6 induces EMT through activating STAT3/SMAD3 in NSCLC.

To explore whether Gankyrin could be regulated by TGF-β1 in NSCLC, Sk-lu-1 cells were exposed to TGF-β1 stimulation for 3 days (concentration from 0–10 ng/ml). p-SMAD3 and Gankyrin were evaluated by Western blot assay. The results showed that the expressions of p-SMAD3 and Gankyrin were higher increased along with TGF-β1 concentration, which indicated that Gankyrin could be regulated by TGF-β1 (Figure 7A). To further explore whether Gankyrin could be regulated by TGF-β1 through p-SMAD3 in NSCLC, we applied siRNA transduction to knockdown the SMAD3 expression in Sk-lu-1 cell lines, then the cells were exposed to TGF-β1 stimulation for 3 days (concentration from 0–10 ng/ml), and the p-SMAD3 and Gankyrin were evaluated by Western blot assay. The results showed that there were not any changes on p-SMAD3 and Gankyrin expressions when siRNA-SMAD3 Sk-lu-1 cell lines were exposed to TGF-β1 stimulation, which indicated that Gankyrin could be regulated by TGF-β1 through p-SMAD3 (Figure 7B). As we have shown that Gankyrin could be regulated by TGF-β1 in NSCLC, and TGF-β1 absolutely could induce EMT, we investigated whether Gankyrin also participated in the TGF-β1-induced EMT process. Interestingly, the data showed that the Gankyrin overexpression did markedly induce EMT with TGF-β1; On the other hand, when Gankyrin expression in Sk-lu-1 pCDNA3.1-Gankyrin cells was knockdown, the TGF-β1-induced EMT phenotype reduced rapidly, which further supported that Gankyrin participated in the TGF-β1-induced EMT process (Figure 7C). In order to strongly confirm this result, we silenced Gankyrin expression in Sk-lu-1 pCDNA3.1-Gankyrin cells and found that silencing Gankyrin could inhibit phosphorylation of STAT3 as well as EMT induced by TGF-β1 (Figure 7D).

To our knowledge, IL-6 is a direct upstream mediator of p-STAT3 in many tumors. Here, we try to investigate whether p-STAT3 was activated by Gankyrin via IL-6 production. IL-6 protein in the conditioned medium of the cultured Sk-lu-1 cells was examined by enzyme-linked immune sorbent assay (ELISA). The results showed that Gankyrin and IL-6 protein level was significantly increased in Gankyrin over-expressed Sk-lu-1 cell line when compared with the control cell line (Figure 7E). These results directly proved that Gankyrin could increase IL-6 expression, and p-STAT3 might be activated by Gankyrin via IL-6 production.

To investigate which molecules played key roles in the Gankyrin-induced EMT process, kinds of downstream molecules such as AKT/p-AKT, STAT3/p-STAT3, SMAD3/p-SMAD3 were evaluated by Western blot assay. The expression levels of p-AKT, p-STAT3 and p-SMAD3 were significantly increased in Gankyrin over-expressed Sk-lu-1 cells, while AKT, STAT3 and SMAD3 levels were not changed at all (Figure 7F). To further explore whether p-AKT was associated with the Gankyrin induced EMT process, inhibitor were used. When p-AKT was inhibited, we found that there was not any change on EMT-related biomarkers, p-STAT3 and p-SMAD3 expressions (Figure 7G). These data suggested that AKT activation was not correlated with Gankyrin-induced EMT.

To further explore whether Gankyrin could induce EMT through p-STAT3/ p-SMAD3 or p-SMAD3/p-STAT3 in NSCLC, we applied siRNA transduction to knockdown the STAT3 and SMAD3 expression in Sk-lu-1 cell lines, respectively, then detected the EMT-related biomarkers and p-AKT, p-STAT3 and p-SMAD3 expressions by Western blot assay. Interestingly, when p-STAT3 or p-SMAD3 was decreased, EMT-related biomarkers E-cadherin was significantly increased, whereas Vimentin and Twist1 were significantly decreased, accompanied with down-regulated levels of p-AKT, p-SMAD3 and p-STAT3 (Figure 7H–7I). These data suggested that Gankyrin could activate p-STAT3 or p-SMAD3, forming a closed circle, to induce the EMT. To further verify the role of IL-6 on EMT process, we added exogenous IL-6 (2 ng/L) into the medium of the cultured Sk-lu-1 cells, The results showed that IL-6 could significantly increased the level of Vimentin, Twist1, p-STAT3 and p-SMAD3, and decreased the level of E-cadherin (Figure 7J).

**Figure 8:**
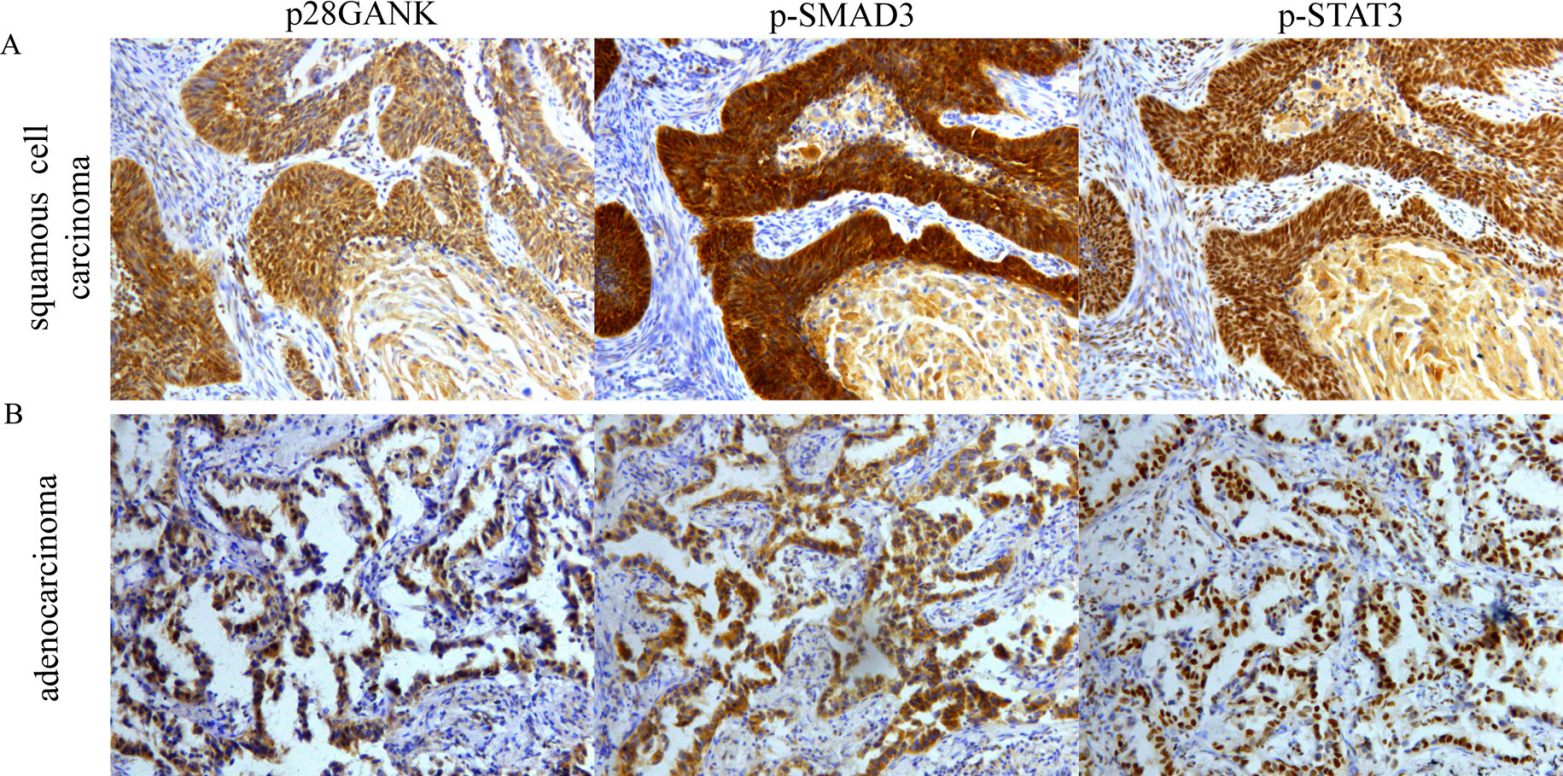
Correlation of Gankyrin expression with p-SMAD3 and p-STAT3 in NSCLC tissues using IHC (**A**) Correlation of Gankyrin expression with p-SMAD3 and p-STAT3 from the same SCC tissues. (**B**) Correlation of Gankyrin expression with p-SMAD3 and p-STAT3 from the same ADC tissues.

Finally, the percentage of positive expression of p-SMAD3 and p-STAT3 of Gankyrin-positive tumors and Gankyrin-negative tumors in 98 NSCLC tissues were shown in Table 3. Correlation analysis showed that Gankyrin expression in NSCLC tissues had positive correlations with the expression of p-STAT3 (r = 0.322, *P* = 0.002) and also had positive correlations with the expression of p-SMAD3 (*r* = 0.346, *P* = 0.001). IHC assay was used to analyze the correlations among Gankyrin, p-SMAD3 and p-STAT3. Representative microphotographs of the expression of Gankyrin, p-SMAD3, p-STAT3 from the same ADC patient and the same SCC patient are shown in Figure 8A–8B. Taken together, these data strongly supported that Gankyrin-promoted EMT and metastasis was partially due to IL-6/p-STAT3 and TGF-β1/p-SMAD3 pathway activation, which forming a closed circle through Gankyrin.

**Table 3:** Correlation of Gankyrin expression with p-SMAD3 and p-STAT3 in all NSCLC tissues

NSCLC tissues	Gankyrin expression		Correlation coefficient	*P*-value
	Positive (%)	Negative (%)		
**P-SMAD3**			*r* = 0.346	0.001
Positive (%)	42	5		
Negative (%)	30	21		
**P-STAT3**			*r* = 0.322	0.002
Positive (%)	37	4		
Negative (%)	35	22		

## DISCUSSION

It has been established that Gankyrin plays critical role in the development and progression of many types of human cancer [28–32, 22]. We also showed that Gankyrin was aberrantly expressed in NSCLC. In our previous study, we revealed that compare to the corresponding normal tissues, Gankyrin is significantly higher expressed in NSCLC tumor tissues. And we found that Gankyrin over-expression was significantly associated with lymphatic metastasis, TNM stage and poor prognosis, and also was an independent prognostic factor of NSCLC [20]. All those results strongly suggested that Gankyrin might represent a biologically cancer exhibiting highly-malignant clinical behavior.

It is well known that the influence of oncoprotein on the prognosis of patients with tumor mainly dependent on two aspects. The first, oncoprotein can promote tumor growth by various molecular pathways to influence the patients’ survival; Second, oncoprotein can promote tumor metastasis to influence patients’ prognosis. Gankyrin, as an important oncoprotein, has been shown to affect the prognosis of patients with lung cancer, but it is unknown that how Gankyrin will influence the prognosis of patients with lung cancer. Previous study reported that, in lung cancer, increased Gankyrin expression was required for the constitutive activation of Akt and tumorigenesis in those who have ras mutation [33], which indicated that Gankyrin might affect the prognosis of NSCLC partially by promoting tumor growth through activating Akt signal. On the other hand, in our study, the correlation analysis also referred that the Gankyrin over-expression was significantly associated with lymphatic metastasis of NSCLC [20]. So we hypothesized that Gankyrin might also influence the patients’ prognosis by promoting NSCLC metastasis. Our data showed that Gankyrin indeed accelerated the migratory and invasive ability of NSCLC *in vitro*, and these results supported our hypothesis.

Next in order to further explore potential mechanisms of Gankyrin promoting NSCLC metastasis, we analyzed the relationship between Gankyrin and the EMT-related biomarkers. As far as we known, more and more studies found that EMT could promote carcinoma invasion and metastasis [24]. And recently several studies have shown that EMT was associated with the acquisition of the malignant characteristics of NSCLC cells, and also was an initiation step in tumor metastasis [34–38]. On the other hand, our previous study found that the function of Gankyrin on prognosis was apparent in early stages NSCLC (p-TNM I/II), while not obvious in advanced stage (p-TNM III/IV) patients. So we guessed that Gankyrin might accelerate NSCLC metastasis through participating in the EMT process. The results showed that Gankyrin could induce EMT in NSCLC cell lines, and Gankyrin expression was negatively correlated with E-cadherin, while positively correlated with Vimentin and Twist1 expression in NSCLC tissues. These data strongly supported that Gankyrin was associated with EMT in NSCLC.

The mechanism of EMT is a very complex network and remains to be elusive [39]. However, TGF-β1/p-SMAD3 pathway is considered to be a canonical pathway inducing EMT in many tumors, including lung cancer. And some EMT-related oncoprotein or anti-oncoprotein were proved to mediate EMT by accelerating or attenuating the TGF-β signaling pathway [40]. In the present study, we found that Gankyrin-induced EMT was also linked with TGF-β1/ p-SMAD3 signal. Our data also suggested that Gankyrin was a downstream molecule of TGF-β1/ p-SMAD3 signaling pathway. In selected lung cancer cell lines, persistently activated or tyrosine-phosphorylated STAT3 is partially due to IL-6 induction, and IL-6/p-STAT3 pathway activation is confirmed to be critical for lung cancer progression [41]. In our study, we found that Gankyrin could up-regulate the IL-6 level, and then promote EMT by activating p-STAT3. Some previous study indicated that IL-6/STAT3 signal was required for TGF-β-induced EMT process in lung cancer cells [42], and the crosstalk between the IL-6/STAT3 and TGF-β/p-SMAD3 signaling pathway did exist in certain conditions [43, 44]. However, we are not clear if there is any interaction. In our study, the results indicated that the crosstalk was partially dependent on Gankyrin, and Gankyrin was a key molecule to sustain EMT phenotype till the cells localized in a new metastatic site even without the continuous IL-6 and TGF-β stimulation. Taken together, our findings were summarized in a model (Figure 7K) to propose that Gankyrin promotes EMT and metastasis in NSCLC through forming a closed circle with IL-6/p-STAT3 and TGF-β/p-SMAD3 signal pathway.

In addition, there were some shortcomings presented in this study. First, the sample size is limited. Second, we did not investigate the role of Gankyrin on metastasis *in vivo*, which are very important for oncoprotein validation. Third, there are some other EMT related biomarkers; we just chose the E-cadherin, Vimentin and Twist1 for testing. It will be more convincing if other indicators shall be detected in our experiment. To compensate for these shortcomings, we intend to carry out further multi-center clinical studies, expand the sample size and enrich the means of detection, and add the animal study to strength the conclusion that Gankyrin could promote EMT and metastasis in NSCLC.

## MATERIALS AND METHODS

### Patients and tissue samples

Tissue specimens and detailed information were the same as our previous research [20]. Histological classification of tumors was reviewed by pathologists and based on the World Health Organization criteria. All tumors were staged according to the pathological tumor/node/metastasis (p-TNM) classification (7th edition) of the International Union against Cancer [23]. The study protocol was approved by the Regional Ethics Committee for Clinical Research of the Fourth Military Medical University. All patients provided written informed consent for use of their medical records and tissue specimens for research purposes.

### Cell lines and treatments

Non-small cell lung adenocarcinoma cancer cell lines (A549, Sk-lu-1, SPC-A-1, H838), non-small cell lung squamous carcinoma cell lines (H520, Calu-1) and human bronchial epithelial cells (HBE) were preserved in our laboratory. A549, Sk-lu-1, SPC-A-1, H838, H520 and Calu-1 cell lines were maintained at 37°C in a humidified incubator containing 5% CO_2_, in RPMI 1640 with supplemented with 10% fetal bovine serum and 1% penicillin/streptomycin. HBE cell line were maintained at 37°C in a humidified incubator containing 5% CO_2_, in high sugar Dulbecco's Modified Eagle Medium supplemented with 10% fetal bovine serum and 1% penicillin/streptomycin. TGF-β1 (ProSpec, 10 ng/ml)/IL-6 (PeproTech, 2 ng/L) was dissolved in DMSO (Sigma, St. Louis, MO) and stored in small aliquots at –20°C.

### Immunohistochemistry

The tumor samples were fixed with 10% formaldehyde and embedded with paraffin. Sections were sliced up at 4 μm thickness, deparaffinized with a series of xylene and rehydrated through a graded series of alcohol. Microwave antigen retrieval was performed at 750 W for 5 min and 450 W for 15 min in citrate buffer (pH 6.0) to enhance the immuno-reactivity. After blocking the endogenous peroxidase activity with 3% hydrogen peroxidase for 30 min, sections were incubated with 5% normal goat serum for 30 min at room temperature to block nonspecific antibody reaction. After washing the tissue samples with PBS three times for 5 min, sections were incubated with the primary antibodies (Gankyrin, 1:50, Santa Cruz Biotechnology, Inc. USA; p-SMAD3, 1:100, Abcam, Cambridge, UK; p-STAT3, 1:100, Cell Signaling, Inc. USA) overnight at 4°C, and incubated with an EnVision™ Detection Kit (DAKO, Denmark) following the manufactures’ instruction. The sections were then reacted with 0.003% 3, 30-diaminobenzidine and counterstained with hematoxylin. To confirm the specificity of the immunostaining, negative controls were obtained by replacing the primary antibody with PBS.

### Evaluation of immunohistochemical staining

Five random fields from each section were viewed under a light microscope (Leica DM4000B, Germany) at × 200 magnification. The expression of protein was scored by multiplication of the percentage of positive tumor cells and the staining intensity [20, 24, 25].

### Plasmid construction and transfection

For Gankyrin overexpression, the whole cDNA sequence of Gankyrin was cloned into the Hind III and Bam HI site of pCDNA(+)3.1 vector (Invitrogen, Carlsbad, CA) and transformed into DH5α competent cells. Positive clones were identified and verified by using restrictive cleavage and sequenced.

H520 and SK-LU-1 cell lines were plated into six-well plates at 5 × 10^5^ cells per well. After 24 h, cells were transfected with 2 μg or 4 μg Gankyrin expression vector using Lipofectamine 2000 (Invitrogen, Carlsbad, CA) according to the manufacturer’ s instruction. Stable clones were isolated after selection with 800 μg/ml of G418 for 1 week, and then 400 μg/ml of G418 for 3 weeks.

### Lentivirus vectors for Gankyrin RNA interference

To observe the effect of inhibiting Gankyrin on human NSCLC cells, we employed the newly developed lentivirus-delivered RNA interfering technique. hU6-MCS-CMV-EGFP-Lentivirus (GeneChem, China) was used to express small interfering RNAs (siRNAs) targeting the Gankyrin sequence (Genbank No.5716). A non-targeting sequence was used as a lentivirus negative control (NC). Targeted oligonucleotide sequences were: siRNA-1: GGTTGGTCTCCTCTTCATA; siRNA-2: CAGCTTGGATTTATTCTTA; siRNA-3: GTTACTTGT TCGAAGCTTA and TTCTCCGAACGTGTCACGT for the lentivirus negative control. Preliminary experiment results showed that siRNA-1significantly could inhibited Gankyrin expression, therefore, siRNA-1 was selected as an optimal siRNA for following experiments. NSCLC cell lines, Calu-1 (SCC) and H838 (ADC), were infected with Gankyrin-siRNA lentivirus and with NC lentivirus. Cells were plated in 6-well plates (5 × 10^4^ cells/well), grown to 60% confluence, and treated with tittered viral supernatant ata multiplicity of infection (MOI) of 20 for 12 h without toxic effect observed. Then, the media was changed to RPMI 1640 medium supplemented with 10% FBS. The interference efficiency of the template was detected by RT-PCR and Western blot analysis. The Calu-1 and H838 cells were transfected with the Gankyrin-siRNA lentivirus or NC lentivirus were designated as Calu-1-siRNA or Calu-1-NC and H838-siRNA or H838-NC, respectively. Non transfected cells were also included as a positive control and designated Calu-1-Control or H838-Control.

### Purification of total cellular mRNA and semi-quantitative RT-PCR

Total RNA was extracted from the fresh tissue specimen of NSCLC patients using E.Z.N.A.^®^ Total RNA Kit I (Omega Bio-TekInc., Georgia, USA) according to the manufacturer's protocols. Reverse transcription of total cellular RNA was performed using a RevertAid First-Strand cDNA Synthesis Kit (Thermo scientific, Vilnius, Lithuania). cDNA was subjected to PCR for 35 cycles of amplification using an cDNA PCR kit (CWbiotech, Peking, China). Each PCR cycle consisted of a denaturation step for 45 s at 95°C, an annealing step for 35 s at 62°C, and an extension step for 50 s at 72°C. The PCR products were separated on 1.0% agarose gel and visualized by ethidium bromide staining. β-actin mRNA was used as an internal control for semi-quantitative analyses of Gankyrin mRNA. The PCR primers used for Gankyrin were 5′-AGCAGCCAAGGGTAACTTGA-3′ as the forward primer and 5′-TACTTGCTCCTTGGGACACC-3′ as the reverse primer; and for β-actin, 5′-CTCCATCCTGGCCT CGCTGT-3′ was used as the forward primer and 5′-GCTGTCACCTTCACCGTTCC-3′ as the corresponding reverse primer.

### Protein isolation and western blot

Cytoplasmic and nuclear protein were extracted from cells using RIPA lysate (P0013B, Beyotime) and Western Blot were performed using anti-Gankyrin specific monoclonal antibody (Santa Cruz Biotechnology, Inc. USA), p-SMAD3 monoclonal antibody (Ser423/425, Abcam), p-STAT3 monoclonal antibody (D3A7, Cell Signaling) and the β-actin specific polyclonal antibody (CW0097, CWbiotech).

Protein concentrations were determined using the BCA assay kit (Pierce). Equivalent amounts of each protein sample were mixed with loading buffer (CW0027A, CWBIO), heated at 65°C for 30 min, and subjected to SDS-PAGE using 12% separation gel and 5% spacer gel. Then protein was transferred to a PVDF membrane (Solarbio) by electro blotting (Bio-RAD). The membrane was blocked for 3 hours at room temperature in TBST (25 mM Tris/HCl, pH 7.5, 150 mM NaCl, 0.1% Tween 20) containing 5% nonfat dry milk. The membranes were incubated overnight at 4°C in 1:200 dilution of anti-Gankyrin monoclonal antibody, 1:1000 dilution of SMAD3 monoclonal antibody, 1:2000 dilution of p-SMAD3 monoclonal antibody, 1:1000 dilution of STAT3 monoclonal antibody, 1:2000 dilution of p-STAT3 monoclonal antibody, 1:500 dilution of AKT monoclonal antibody, 1:1000 dilution of p-AKT monoclonal antibody, 1:500 dilution of E-cadherin monoclonal antibody, 1:2000 dilution of Vimentin monoclonal antibody, 1:300 dilution of Twist1 monoclonal antibody, 1:500 dilution of IL-6 monoclonal antibody and 1:2500 dilution of β-actin polyclonal antibody with WB Antibody Diluent (P0023A, Beyotime), washed six times in TBST, and incubated for 35 min in a 1:5000 dilution of goat anti-rabbit Ig-HRP (EK020, Zhuangzhibio) with WB Secondary Antibody Diluent (P0023D, Beyotime). Immunoreactive bands were revealed by the enhanced chemiluminescence system (Santa Cruz Biotechnology, Santa Cruz, CA). Pictures were photographed and analyzed by Gel Dox XR system (Bio-Rad, Philadelphia, PA).

### Scratch wound healing assay

Cell migration was measured using a scratch assay determined, as described previously [26]. After 5 days of lentiviral infection, both transfected and untreated Calu-1 and H838 cells were harvested and their 5 × 10^5^ cells were plated in 6-well plates. Cells were incubated overnight. After incubation, the plates yielded cells of 80% confluence, the monolayer was scraped in a straight line to create a “scratch” using a 200 μL pipette tip. After removing debris and adding fresh media containing no FBS, cells were photographed at 0 h, 24 h, and 48 h. The migration distance was measured and assessed using image J software at 3 different sites from each wound area of scratch, at each time point. The healing degree was calculated by cell relative migration area for each treatment.

### Transwell invasion and migration assay

To further examined the cell invasion and migration, the transwell invasion and migration was performed using 8 μm pore size transwell chambers (Corning, USA) *in vitro* following the manufacturer's instructions. In brief, the matrigel (5 mg/ml, Corning, USA) was diluted into 1 mg/ml in ice-cold RPMI 1640 medium supplemented with 10% FBS. An aliquot of 200 μL diluted matrigel was added to the upper transwell chambers and incubated at 37°C for 4 h for gelling. A total of 1 × 10^5^ cells in 400 μL media supplemented with no FBS were plated in the upper chamber and 600 μL RPMI 1640 medium supplemented with 10% FBS was covered on the bottom chambers as chemo attractant. After incubation at 37°C for 48 h, the non-invasive cells in the top surface were carefully removed with a cotton swab. The invasive cells that had traversed to the bottom surface were fixed in dehydrated alcohol for 30 min and stained with 4 mg/ml crystal violet for 10 min. To quantify the traversed cells, cell counting was obtained by photographing 5 random fields under microscope at 400× magnification [27]. The migration assay was performed in a similar strategy with chamber membrane without coating with matrigel.

### ELISA

Supernatants from NSCLC cells were collected and IGF1 ELISA was subsequently performed using IGF1 ELISA Kit and following the manufacturer's instructions.

### Immunofluorescence (IF)

Cells were fixed in 4% paraformaldehyde for 30 min and blocked in 0.5% Trion X-100 for 15 min. The cells were washed 3 times (5 min for each) with PBS after each step. The tumor samples were fixed with 10% formaldehyde and embedded inparaffin. Sections were sliced at 4 μm thickness, deparaffinized with a series of xylene washes, and rehydrated through a graded series of alcohol rinses. Microwave antigen retrieval was performed at 750 W for 5 min and 450W for 15 min in 0.1 M citrate buffer (pH 6.0) to enhance the immunoreactivity. To block the endogenous peroxidase activity, all the cells and tumor samples were incubated in 3% hydrogen peroxidase at room temperature for 30 min and washed with PBS three times for 5 min. All the cells and tumor samples were incubated with 10% normal goat serum for 30 min at room temperature to block nonspecific antibody reaction, followed by incubation in a humidified chamber overnight at 4°C with anti-Gankyrin rabbit polyclonal antibody (1:50, Santa Cruz Biotechnology). After an additional series of washes, the samples were stained with goat anti-rabbit (Cy3, Zhuangzhi bio) or were stained with goat anti-rabbit (Alexa Fluor 488, Zhuangzhi bio) at 37°C for 4 min, then each sample was washed with PBS three times, for 5 min. Then, the samples were incubated with 4′6-diamino-2-phenylindole (DAPI) antibody (1:160, Abcam) for 5–10 min at room temperature. After the final washing, the samples were mounted in 50% glycerol (in PBS) and visualized under a fluorescence microscope (Leica DM4000B, Germany).

### Statistical analysis

Each experiment was performed in triplicate. Bands from Western blot were quantized using Quantity One software (Bio-Rad, USA). Relative protein levels were calculated relative to the amount of β-actin respectively. All statistical analyses were performed with SPSS 18.0 software (SPSS, Inc., Chicago, IL). All values in the text and figures are expressed as the mean ± SD of these observations. Student's *t-test* was used for raw data analysis. A *P-value* < 0.05 was considered statistically significant.
